# Possible Involvement of Skin‐Resident Memory T Cells in Refractory Chronic Alopecia Areata

**DOI:** 10.1111/exd.70212

**Published:** 2026-01-19

**Authors:** Reiko Kageyama, Taisuke Ito, Kazuo Kurihara, Toshiharu Fujiyama, Tetsuya Honda

**Affiliations:** ^1^ Department of Dermatology Hamamatsu University School of Medicine Hamamatsu Japan

**Keywords:** alopecia areata, JAK inhibitor, resident memory T cell, treatment‐refractory

## Abstract

Alopecia areata is a typical skin disease with unmet needs. So far, it has been understood that the main cause of the intractability of chronic cases is the decrease in inflammatory cell infiltration and falling into the telogen‐like phase. However, in some cases, even in long‐term chronic cases, inflammatory cell infiltration can be seen, so that we speculate that the long‐term persistence of these perifollicular cells may be the reason for the lack of improvement as skin resident memory T (T_RM_) cells. To investigate the presence of T_RM_, nine treatment‐resistant chronic AA patients and 5 acute AA patients were employed for staining with CD69 and CD103 as markers for identifying skin T_RM_ cells. This study revealed the number of CD8^+^CD103^+^ T and CD69^+^CD103^+^ T cells tended to increase with increasing disease duration and refractoriness. In one case of AA refractory to conventional treatment, an oral JAK inhibitor (JAKi) showed dramatic efficacy with a reduction in the number of infiltrating CD103^+^ cells, including CD8^+^CD103^+^ T and CD69^+^CD103^+^ T cells. These results suggest that refractory cases in the chronic phase tend to have more infiltrating skin T_RM_ cells, and JAKi may be effective in the refractory cases of chronic AA.

## 1 Background

Alopecia areata (AA) is a cell‐mediated autoimmune disease in which the immune privilege (IP) of hair follicles (HFs) collapses and HF autoantigens are recognised by autoreactive NKG2D^+^CD8^+^ T cells that produce large amounts of interferon (IFN)‐γ [1, 2, 3]. However, the detailed pathomechanism remains unclear [4, 5]. While some cases of AA resolve spontaneously within a few months, there are also intractable cases that are resistant to all treatments and last for more than 10 years. In the chronic phase of AA, HFs become miniaturised like telogen hair, and perifollicular inflammatory cellular infiltrate is greatly reduced [5]. This reduction of inflammatory cells has been considered to be a reason for the less efficacy of anti‐inflammatory drugs, such as corticosteroids, and contact immunotherapy to modulate the immune balance. However, in reviewing the histopathology of refractory chronic AA cases, we noticed that although the degree of cellular infiltration around HFs was indeed reduced, there were inflammatory cells that infiltrated and adhered to the telogen‐like HFs in the AA lesions. Our previous study showed that CD8^+^ T cells persisted around hair bulbs, whereas infiltrating CD4^+^ T cells decreased in chronic‐phase AA lesions [6]. In psoriasis vulgaris, the presence of resident memory T (T_RM_) cells at recurrence sites might be associated with its chronicity [7]. Some T_RM_ cells migrate into tissues and remain there without returning to the circulation, and several studies have suggested a pathological role for skin T_RM_ cells in cutaneous inflammatory diseases, such as psoriasis [8, 9, 10, 11]. Skin T_RM_ cells expressing the tissue‐retention markers CD103 and CD69 are considered to likely be responsible for inducing relapse [12, 13]. In AA, tissue T_RM_ cells have been hypothesized to be involved in AA pathogenesis, as increased levels of these cells have been observed in AA lesions in only one review paper [14]. In another paper, it is reported that a histopathological examination of chronic AA patients showed that the number of CD8^+^CD69^+^ T_RM_ cells around HFs increased with the severity of histopathological grades [15]. However, the relative contribution of skin‐resident T cells over those newly infiltrated in the pathogenesis of AA is unclear [16]. In chronic and resistant cases, a large number of inflammatory cells do not infiltrate around the hair follicles; however, a dense lymphocytic infiltrate is often observed in and along the perifollicular epithelium. We hypothesized that the long‐term persistence of these perifollicular cells in the periphery of the HFs may be responsible for the lack of improvement in alopecia symptoms, and that it may involve cutaneous T_RM_ cells.

## 2 Questions Addressed

In order to investigate the role of skin T_RM_ cells in patients with long‐standing refractory AA, we performed immunohistochemical studies of CD8^+^ skin T_RM_ cells using lesional skin samples from the patient with treatment‐resistant chronic AA and acute AA.

## 3 Experimental Design

Lesional skin samples were obtained from nine patients with chronic AA who had not responded to various treatments, including contact immunotherapy, oral corticosteroids and intradermal corticosteroid injections for more than 6 months (Table 1). For comparisons, lesional skin samples were also obtained from five patients with acute‐phase AA within 6 months of disease onset. The formalin‐fixed and paraffin‐embedded lesional skin samples were deparaffinised and stained with haematoxylin and eosin. Next, we performed immunohistochemical studies to examine CD8^+^ skin T_RM_ cells in lesional skin samples from treatment‐resistant chronic AA patients and acute AA patients. CD69 and CD103 were used to identify skin T_RM_ cells. Unlike other phenotypic markers of T_RM_ cells, these markers have been associated with alopecia areata in more studies [14]. Multiple immunostaining was performed with fluorescent antibodies as previously described [12]. Paraffin‐embedded skin samples were deparaffinised and were treated in an autoclave with 0.01 M sodium citrate buffer (pH 6.0). Slides were blocked with 1% FBS in 10 min, then stained with primary antibody against human antigens (diluted 1:100 with PBS), including CD4, CD8, CD103, CD69, FABP4, NKG2D and IL‐15 for 2 h (Table S1), followed by staining with secondary antibody (diluted 1:100 with 1% FBS), including Alexa Fluor 488 anti‐rabbit IgG (Abcam) and Alexa Fluor 594 anti‐mouse IgG (ThermoFischer Scientific Walham, MA) for 1 h. They were rinsed in PBS each time, then stained with DAPI for 1 min. Digital images were obtained using NanoZoomer (Hamamatsu Photonics, Hamamatsu, Japan), and the image data were used for objective quantitative analysis by image cytometry using StrataQuest image analysis software (Tissue Gnostics, Vienna, Austria) [7]. In the image analysis, the percentage of infiltrating cells in the region of connective tissue sheath (CTS) around the HF was calculated [17]. The same method was also used to evaluate the lesional skin before and after treatment in a case of refractory AA that had improved with an oral JAK inhibitor (JAKi). The Mann–Whitney *U*‐test with Bonferroni correction followed by the Kruskal‐Wallis *H*‐test was used to assess the statistical significance of differences between mean values. *p* < 0.05 was considered to be statistically significant. This study was conducted in accordance with the tenets of the Declaration of Helsinki, and the study protocol was approved by the Ethics Committee of Hamamatsu University School of Medicine (approval numbers 17–089 and 18–026).

**TABLE 1:** Summary of the alopecia areata cases that were analysed.

Types of AA		Age (y/o)	Sex	Disease duration	Response to treatment
Acute phase	AA totalis: 5	Avg. 41.6 (21 to 76)	M: 3 F: 2	Avg. 3 months (2 to 4 months)	Responder: 5 (Contact immunotherapy, oral corticosteroids, corticosteroid pulse therapy)
Chronic phase	AA multiplex: 1 AA totalis: 2 AA universalis: 6	Avg. 48.1 (12 to 81)	M: 2 F: 7	Avg. 64 months (10 to 180 months)	Responder: 3 (Dupilumab, JAKi) Non‐responder: 6

## 4 Results

**FIGURE 1:**
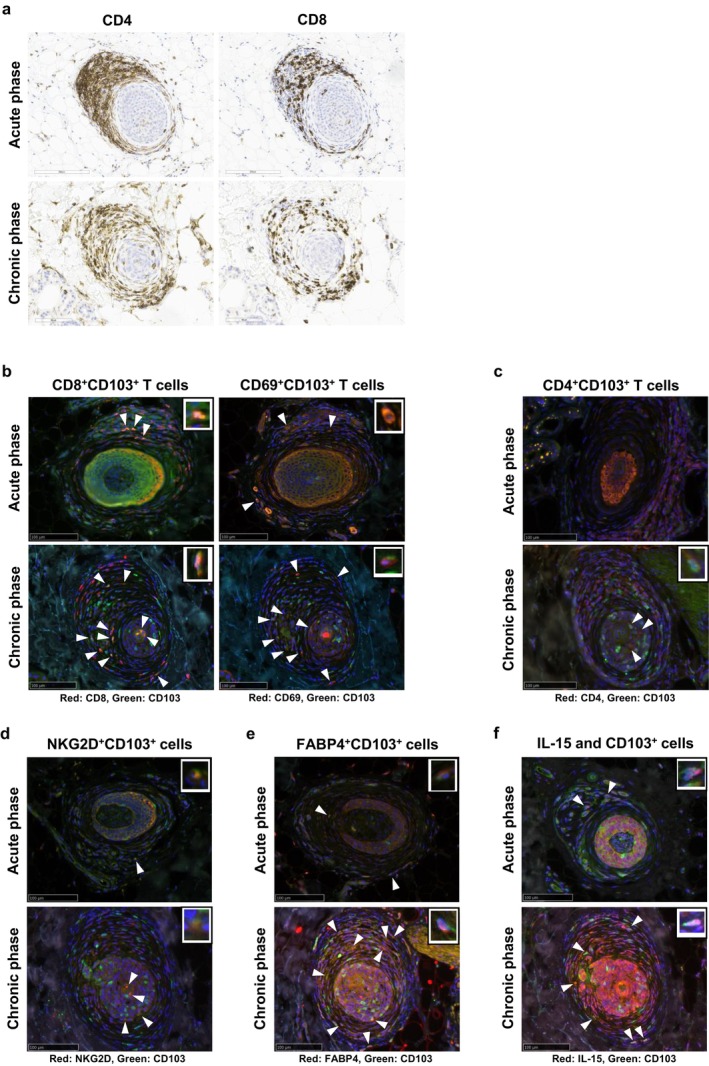
Histopathology and fluorescent immunostaining of lesional skin in alopecia areata (AA) cases. A greater infiltration of CD4^+^ T cells than CD8^+^ T cells is observed around hair follicles in the acute AA lesions, and the ratio of infiltrating CD4^+^ T cells to CD8^+^ T cells is lower in the chronic AA lesions (a). In the chronic AA lesions, CD8^+^CD103^+^ T and CD69^+^CD103^+^ T cells are numerous (CD8 or CD69: Red, CD103: Green, merged cell: Yellow (arrow)), and they infiltrate around the hair bulb and within the surrounding CTS (b). In chronic AA lesions, infiltration of CD4^+^CD103^+^ T cells (CD4: Red, CD103: Green, merged cell: Yellow (arrow)) is observed within the hair bulb (c). NKG2D^+^ cells (red) are present among the CD103^+^ infiltrating cells (green) (d). In chronic AA lesions, FABP4 + CD103+ T cells are numerous (FABP4: Red, CD103: Green, merged cell: Yellow (arrow)) around the hair bulb and within the surrounding CTS (e). IL‐15 (red) tends to be highly expressed in epithelial cells in the pilocytic area in chronic phase lesions (f).

**FIGURE 2:**
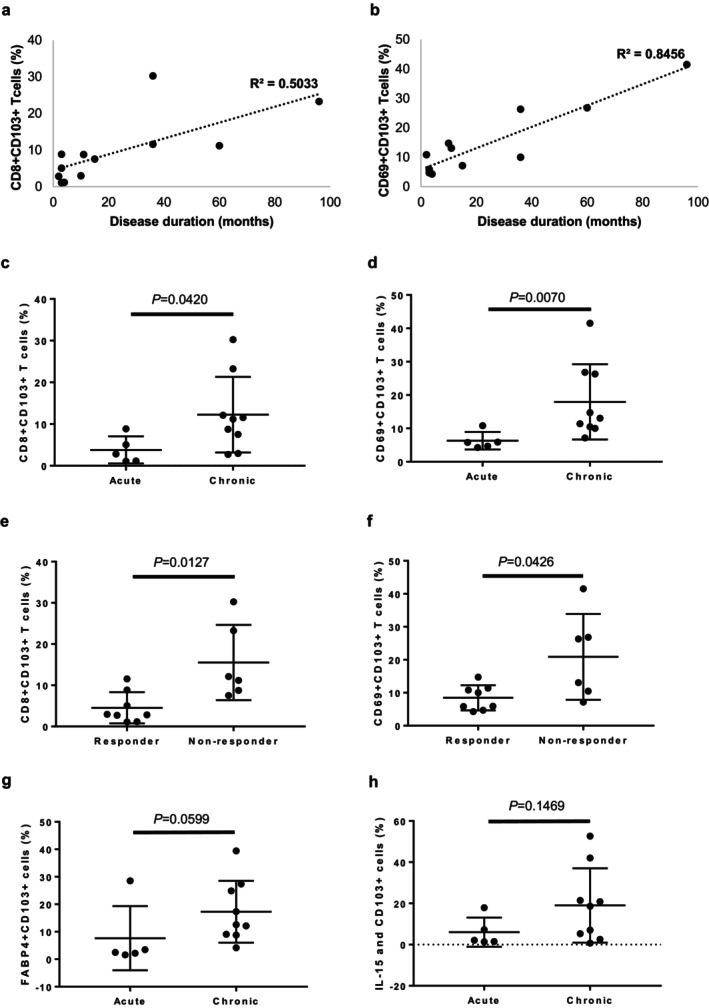
Comparative analysis of skin resident memory T (T_RM_) cells. Relationship between skin T_RM_ cells and disease duration (a, b). Percentage of infiltrating skin T_RM_ cells in the lesional skin of acute AA and chronic AA cases (c, d). Percentage of infiltrating skin T_RM_ cells in the lesional skin of acute AA and chronic AA cases when cases with a SALT score improvement of 70% or more within 36 weeks of treatment initiation are defined as responders (e, f). Percentages of FABP4^+^CD103^+^ T cells and IL‐15 and CD103^+^ cells in acute AA and chronic AA cases (g, h).

Analysis of the inflammatory cell infiltration around HFs in AA lesions revealed that the ratio of CD4^+^ to CD8^+^ infiltrating T cells was approximately 2:1 in acute‐phase lesions, and that the ratio tended to be lower in chronic‐phase lesions (Figure 1a). In the acute‐phase lesions, inflammatory cells infiltrated a relatively large area, not only the perifollicular area; in contrast, in the chronic‐phase lesions, the infiltration was concentrated in the HF CTS and some of the hair matrix (Figure 1a). In most chronic AA lesions, there were numerous CD8^+^CD103^+^ T and CD69^+^CD103^+^ T cells, and they infiltrated around the hair bulb and within the surrounding HF CTS (Figure 1b. CD8 or CD69: red, CD103: green, merged cell: yellow). In addition, infiltration of CD4^+^CD103^+^ T cells within the hair bulb was observed in chronic AA lesions (Figure 1c. CD4: red, CD103: green). Furthermore, NKG2D^+^ cells were present among these CD103^+^ infiltrating cells (Figure 1d. NKG2D: red, CD103: green). When we examined the relationship between the skin T_RM_ cells and disease duration, we found that chronic AA cases with a longer disease duration tended to have more CD8^+^CD103^+^ T cells and CD69^+^CD103^+^ T cells, and that the numbers of these cells differed significantly between the chronic‐phase and acute‐phase lesions (*p* = 0.0420 and *p* = 0.0070, respectively; Figure 2a–d). In addition, we defined responders as cases in which the Severity of Alopecia Tool (SALT) score improved by 70% or more within 36 weeks of treatment initiation. Then, the relationship between skin T_RM_ cells and treatment response were examined. We found that treatment‐resistant AA cases tended to have more CD8^+^CD103^+^ T (*p* = 0.0127) and CD69^+^CD103^+^ T (*p* = 0.0426) cells (Figure 2e,f).

Unlike other memory T cell fractions, skin T_RM_ cells utilise fatty acids as an energy source for ATP synthesis through the uptake and processing of free fatty acids (FFAs), enabling long‐term survival [11]. Namely, fatty acid‐binding protein (FABP) 4 and 5 play critical roles in the maintenance, longevity and function of CD8^+^ T_RM_ cells, suggesting that CD8^+^ T_RM_ cells use exogenous FFAs and oxidative metabolism to survive in tissues and mediate protective immunity [18]. In this study, we found that there tended to be more FABP4^+^CD103^+^ T cells in chronic AA than in acute AA (Figure 1e. FABP4: red, CD103: green. *p* = 0.0599; Figure 2g); in particular, there tended to be more FABP4^+^CD103^+^ T cells in treatment‐resistant AA (*p* = 0.0426). Interleukin (IL)‐15 also plays an important role in the maintenance and proliferation of cytotoxic T cells. In this study, IL‐15 tended to be highly expressed in epithelial cells in the pilocytic area in chronic‐phase lesions, although there was no significant difference when compared to acute‐phase lesions (Figure 1f. IL‐15: red, CD103: green. *p* = 0.1469; Figure 2h).

**FIGURE 3:**
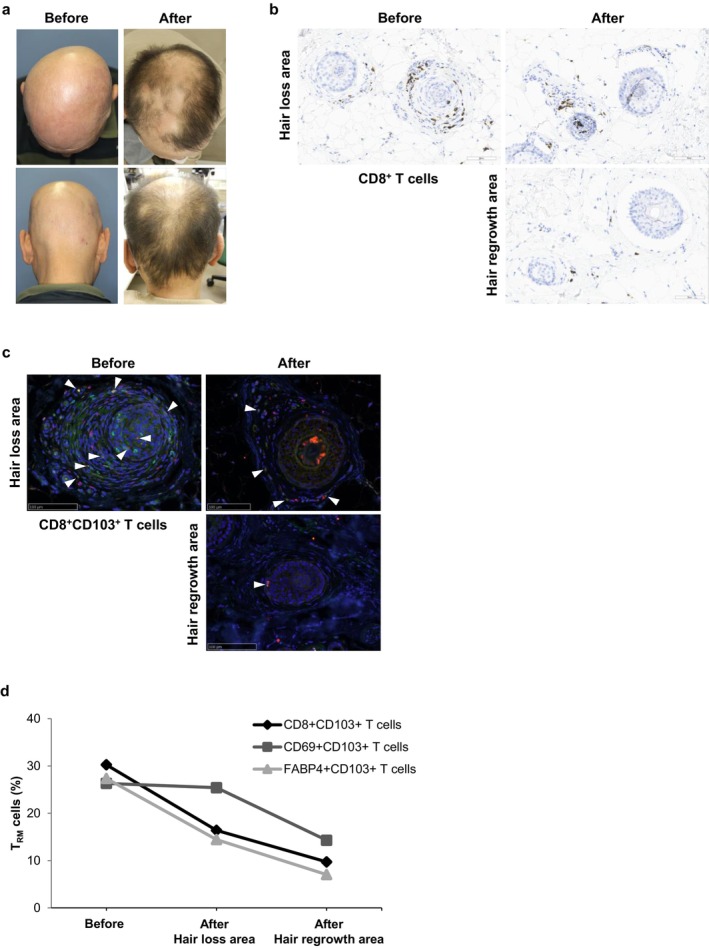
Histology of the lesions before and after treatment with the JAK inhibitor (JAKi). Clinical findings before and after treatment with an oral JAKi (a). Infiltration of CD8^+^ T cells in the hair loss area and the regrowth area before and after JAKi treatment (b). Infiltration of CD8^+^CD103^+^ T cells (CD8: Red, CD103: Green, merged cell: Yellow (arrow)) in the hair loss area and the regrowth area before and after JAKi treatment (c). Percentages of the infiltrating skin T_RM_ cells in the hair loss area and the hair regrowth area before and after JAKi treatment (d).

Baricitinib, an oral JAK1/2 inhibitor, was approved in 2022 for treating patients with severe AA, that is, those with a SALT score of 50 or higher (range, 0 [no scalp hair loss] to 100 [complete scalp hair loss]) [19], and its efficacy has been proven by real‐world data and clinical trials in clinical trials and in real‐world settings [20]. Because baricitinib was effective in a patient who was refractory to oral corticosteroids in the present study, the skin T_RM_ cells in this patient were investigated before and after the baricitinib treatment. The patient was a 54‐year‐old male who had suffered from intractable AA for 3 years and was treated with contact immunotherapy and intermittent oral corticosteroids. However, he developed AA universalis 6 months after the first visit (Figure 3a). He was therefore started on baricitinib, and his SALT score improved from 100% to 34.6% after 5 months (Figure 3a). Histopathological examination before baricitinib treatment revealed somewhat reduced inflammatory cell infiltrates in the perifollicular area, but high inflammatory cell infiltrates, including persistent CD8^+^ T cells, in the CTS (Figure 3b). Histopathology of the hair loss and regrowth areas 5 months after baricitinib treatment revealed a significant reduction in CD8^+^ T cell infiltration, and an even greater reduction in the hair regrowth area when compared to before treatment (Figure 3b). In addition, the comparison of lesional skin T_RM_ cells before and 5 months after starting JAKi treatment revealed that the percentages of CD8^+^CD103^+^ T, CD69^+^CD103^+^ T and FABP4^+^CD103^+^ T cells in the perifollicular area decreased after the JAKi treatment (Figure 3c,d. CD8: red, CD103: green). The proportion of skin T_RM_ cells was decreased more in areas of hair regrowth than in areas of hair loss after starting JAKi treatment (Figure 3c,d).

## 5 Conclusions and Perspectives

This study revealed that there were more CD8^+^CD103^+^ T and CD69^+^CD103^+^ T cells in the chronic AA cases with a longer disease duration than in the acute AA cases. In addition, more CD8^+^CD103^+^ T, CD69^+^CD103^+^ T and FABP4^+^CD103^+^ T cells were present in the treatment‐refractory AA cases than in the treatment‐responsive AA cases. These results suggest that refractory cases of chronic‐phase AA tend to have more T_RM_ cells infiltrating the skin than acute‐phase AA cases. The persistence of skin T_RM_ cells in the treatment‐refractory AA patients despite prior corticosteroid treatment may indicate steroid resistance. Moreover, the presence of NKG2D^+^ cells among the CD103^+^ infiltrating cells suggests that infiltrating skin T_RM_ cells are also AA effector cells. The long‐term persistence of these activated skin T_RM_ cells in the hair bulb and CTS may contribute to the refractoriness of AA. Increased number of FABP4^+^CD103^+^ T cells and higher expression of IL‐15 in epithelial cells of pilocytic area in chronic AA inferred the maintenance of T_RM_ cells by FABP4 and IL‐15 in chronic AA. Although IL‐15 is thought to be a key pathogenic cytokine in AA, Suzuki et al. [21] reported that IL‐15 is a functionally important HF‐IP guardian. It may be related to our finding of some variation in the proportion of IL‐15^+^ T cells in chronic‐phase AA.

In our study, JAKi showed dramatic efficacy in a case of AA refractory to conventional treatments, including contact immunotherapy; JAKi decreased the number of infiltrating CD103^+^ cells, including CD8^+^CD103^+^ T and CD69^+^CD103^+^ T cells. Baricitinib, a JAK1/2 inhibitor, is thought to exert a therapeutic effect on cases that show resistance to treatment by weakening the maintenance and survival of T_RM_ cells through the inhibition of JAK1, which binds to the IL‐15 receptor [22]. JAKi has the potential to eliminate persistently infiltrating skin T_RM_ cells and improve refractory chronic AA cases. It is assumed that newly initiated JAKi treatment may be effective in reducing T_RM_ cell infiltration when infiltration has persisted for a long time in refractory chronic AA cases. However, this study examined only one case, so further analysis with a larger sample size is required to confirm the JAKi therapy's efficacy in reducing T_RM_ cells.

In conclusion, this research suggests that T_RM_ cells contribute to the chronicity and treatment resistance of AA, and it is speculated that T_RM_ cells may become a novel treatment target for chronic AA. In this context, JAKi is suggested as a new treatment option with the potential to eliminate T_RM_ cells, and further case analysis is anticipated.

## Author Contributions

R.K. involved in investigation and writing – original draft. T.I. involved in investigation and writing – review and editing. K.K. involved in methodology and writing – review and editing. T.F. involved in methodology and writing – review and editing. T.H. involved in writing – review and editing. Final approval of the version to be published: R.K., T.I., K.K., T.F., T.H.

## Conflicts of Interest

The authors declare no conflicts of interest.

## Supporting information


**Figure S1:** Comparative analysis of expression of IL‐15 in the epithelium.

**Table S1:** Summary of the antibody reagents used for immunostaining and fluorescence immunostaining.

## Data Availability

The data that support the findings of this study are available from the corresponding author upon reasonable request.
