# Supplementary figures and images for: Possible Involvement of Skin‐Resident Memory T Cells in Refractory Chronic Alopecia Areata
Source: Exp Dermatol. 2026 Jan 19;35(1):e70212. doi: 10.1111/exd.70212 (PMC12816445; doi:10.1111/exd.70212)

## Slide 1
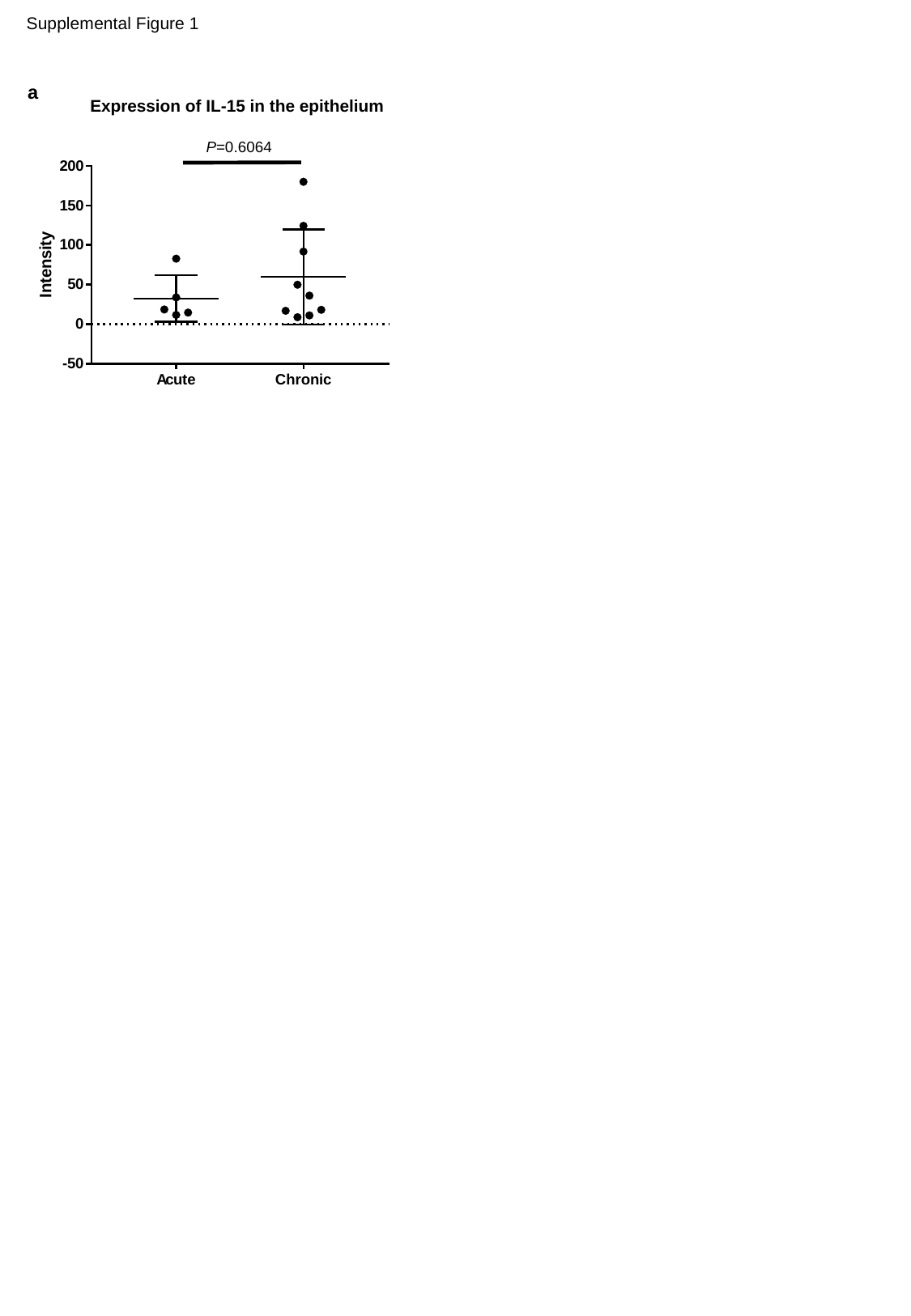

Supplemental Figure 1
a
Expression of IL-15 in the epithelium
P=0.6064

Supplement: Supplementary file 1 — Figure S1: Comparative analysis of expression of IL‐15 in the epithelium. [file EXD-35-e70212-s002.pptx]
