# Supplementary material for: Possible Involvement of Skin‐Resident Memory T Cells in Refractory Chronic Alopecia Areata
Source: Exp Dermatol. 2026 Jan 19;35(1):e70212. doi: 10.1111/exd.70212 (PMC12816445; doi:10.1111/exd.70212)
Supplement: Supplementary file 2 — Table S1: Summary of the antibody reagents used for immunostaining and fluorescence immunostaining. [file EXD-35-e70212-s001.docx]

**Supplemental Table**

S1: Summary of the antibody reagents used for immunostaining and fluorescence immunostaining.

| CD4 | Monoclonal Mouse Anti-Human CD4, Dako, Glostrup, Denmark; 1:100 dilution |
| --- | --- |
| CD8 | Monoclonal Mouse Anti-Human CD8, Dako, Glostrup, Denmark; 1:100 dilution |
| CD103 | Monoclonal Rabbit Anti-CD103 antibody, Abcam, Cambridge, UK; 1:100 dilution |
| CD69 | Monoclonal Mouse CD69 antibody(D-3), Santa Cruz, Texas, USA; 1:100 dilution |
| FABP4 | Monoclonal Mouse A-FABP antibody(B-4), Santa Cruz, Texas, USA; 1:100 dilution |
| IL-15 | Monoclonal Mouse IL-15 antibody(E-4), Santa Cruz, Texas, USA; 1:500 dilution |
| NKG2D | Polyclonal Mouse Anti-NKG2D antibody, Abcam, Cambridge, UK; 1:400 dilution |
